# Improving Contraceptive Service Quality and Accessibility for Adolescents and Youth Through Proprietary Patent Medicine Vendors in Four Nigerian States

**DOI:** 10.9745/GHSP-D-22-00225

**Published:** 2024-05-21

**Authors:** Dorcas Akila, Oluwasegun Akinola, Olukunle Omotoso, Saori Ohkubo, Adewale Adefila, Philemon Yohanna, Nwanne Ikodiya Kalu, Adebusola Oyeyemi, Olubunmi Ojelade, Aisha Waziri, Winifred Kwaknat, Olusola Solanke, Bernard Emonena, Oluwafemi Rotimi, Lisa Mwaikambo, Victor Igharo, Lekan Ajijola, Krishna Bose

**Affiliations:** a The Challenge Initiative, Nigeria Hub, Johns Hopkins Center for Communication Programs, Abuja, Nigeria.; b The Challenge Initiative, Johns Hopkins Center for Communication Programs, Baltimore, MD, USA.; c The Challenge Initiative, Bill & Melinda Gates Institute for Population and Reproductive Health, Baltimore, MD, USA.

## Abstract

**Introduction:**

In Nigeria, health care services and commodities have increasingly been accessed through private sector entities, including retail pharmacies and drug shops (also called proprietary patent medicine vendors [PPMVs]). However, PPMVs cannot provide long-acting or permanent methods, and concerns have been raised about their quality of services and their need to better comply with government regulations. This article describes how The Challenge Initiative’s (TCI) family planning program supported 4 state governments in Nigeria to develop a model to strengthen public-private partnerships between PPMVs and primary health centers (PHCs) to leverage PPMVs to provide adolescents and youth with high-quality contraceptive information, services, and referrals to PHCs.

**Program Description:**

The intervention implemented a hub-spoke model by strengthening the linkages between neighboring PPMVs and large PHCs for delivering contraceptive services to adolescents and youth. The steps in the implementation process included: (1) introducing the intervention to state governments, (2) selecting PPMVs as spokes and high-volume PHCs as hubs, (3) conducting whole-site orientations jointly with PPMV and PHC staff, (4) strengthening referral links between PPMVs and PHCs, (5) implementing supportive supervision and coaching, and (6) strengthening client data management. TCI worked with the state and local ministry of health to improve PPMV operators’ knowledge, attitudes, and skills to deliver adolescent- and youth-friendly services.

**Lessons Learned and Recommendations:**

Implementing the PPMV intervention with state governments and PHCs strengthened the public-private partnership. A functional referral system in Plateau State demonstrated significant success, enabling increased contraceptive choice and adherence to regulations for adolescents and youth. We recommend that the government strengthen the working relationship between PPMVs and PHCs, incorporate PPMVs into the routine supportive supervision of the state health system, and incorporate a referral linkage with PHCs into the design and implementation of PPMV programs.

## Abstract (precis)

Building a collaborative partnership between public and private sector health entities can expand access to and improve the quality of contraceptive information and services provided to adolescents and youth living in urban poor communities.

## Teaser

**Key Findings:**

- Proprietary patent medicine vendors (PPMVs) serve as a major source of family planning services and short-term contraceptive methods, particularly for adolescents and youth aged 15–24 years. However, concerns about their quality of services, compliance with national regulations, and low rates of referrals to primary health centers (PHCs) for long-term contraceptive methods posed limitations to meeting adolescents’ and youth’s sexual and reproductive health needs.
- To expand access to quality contraceptive services for adolescents and youth and increase contraceptive uptake to methods not dispensed by PPMVs, The Challenge Initiative aimed to build a collaborative partnership between PPMVs and PHCs to improve PPMVs’ referrals to PHCs and compliance with national guidelines.
- Strengthening the partnership between PPMVs and the state public health system resulted in significant increases in contraceptives dispensed in the 193 participating PPMVs.

**Key Implications:**

- The state health system can design and implement a robust partnership with PPMVs to increase access and quality contraceptive services for adolescents and youth.
- State governments should continue to strengthen collaboration with PPMVs through national, state, and local associations to maintain adolescent and youth sexual and reproductive health service quality and referrals.

## BACKGROUND

In recent decades, Nigerians have increasingly accessed health care services and commodities through private sector entities, including retail pharmacies and drug shops (also known as chemists or proprietary patent medicine vendors [PPMVs] in Nigeria).^1^^,^^2^ PPMVs are Nigerians’ main source of medicine for acute conditions.^3^ They are also significant providers of modern contraceptive methods, with 41% of modern method users in Nigeria reporting the private sector as their source of contraception.^4^ Moreover, a 2015 national contraceptive outlet study documented that 86% of private sector outlets stock contraceptives and provide family planning (FP) services, and of these, 72% are PPMVs.^5^^,^^6^

There are an estimated 200,000 PPMVs in Nigeria that are concentrated in poor urban settings. Overall, in sub-Saharan Africa, it is estimated that up to 70% of all urban residents may live in informal settlements,^7^ and in Nigeria, this is estimated to be 54%.^8^ Few efforts have been documented that address increasing intra-urban inequalities. The situation is further complicated by poverty, population density, and a lack of access to quality services, including FP services, resulting in poor reproductive, maternal, and child health indicators.^9^^–^^11^ Moreover, in urban areas and informal settlements, primary health care structures are often lacking, making the PPMVs more accessible, particularly in Nigeria where they outnumber government primary health centers (PHCs) by a 6-to-1 ratio.^12^^,^^13^

Youth often prefer going to pharmacies and PPMVs over government-owned PHCs for modern short-term contraceptive methods^2^^,^^6^ and contraceptive information and counseling^14^^,^^15^ because PPMVs are located nearby, have long operating hours including weekends, and provide confidential services.^2^ Given the sociocultural bias associated with nonmarital and premarital sex, especially among adolescents and unmarried youth, PPMVs are considered a safe haven for the provision of discreet contraceptive services.^16^ Between October and December 2019, a household survey of all 7,003 adolescents and youth in 4 states (Edo, Niger, Ogun, and Plateau) on adolescent and youth sexual and reproductive health (AYSRH) showed that 74% of respondents who reported using modern contraceptive methods sourced them from the private sector—PPMVs 47%, pharmacies 20%, shops 5%, and private hospitals 2% (Table 1 and Table 2).

**TABLE 1.** Adolescent and Youth Sexual and Contraceptive Experience in Four States in Nigeria

	**No. (%)**
States	
Edo	1,739 (25)
Niger	1,730 (24)
Ogun	1,742 (25)
Plateau	1,792 (26)
Total	7,003
Have you ever had sex?	
Yes	3,587 (51)
No	3,416 (49)
Total	7,003
Had sex in the past 3 months?	
Yes	1,930 (54)
No	1,657 (46)
Total	3,587
Currently using any contraceptive method?	
Yes	1,172 (33)
No	2,415 (67)
Total	3,587
Currently using modern contraceptive method?	
Yes	976 (100)
Source of current modern method	
Private total	722 (74)
Proprietary patent medicine vendor	454 (47)
Pharmacy	194 (20)
Shop	52 (5)
Private hospital	22 (2)
Government hospital	160 (16)
Family and friends	16 (2)
Community health worker	11 (1)
Other	39 (4)
Don’t remember	28 (3)

Source: The Challenge Initiative Background Household Survey, 2019.

**TABLE 2.** Source of Current Modern Contraceptive Method for Youth and Adolescents, by State

**State**	**Government Hospital, %**	**Private Hospital, %**	**Community Health Worker, %**	**Pharmacy, %**	**Chemist, %**	**Shop, %**	**Family/ Friends, %**	**Others, %**
Edo	9.4	2.7	1.3	23.3	59.6	0.5	0.5	2.7
Niger	26.9	2.3	0.7	15.1	42.7	1.2	1.3	9.8
Ogun	15.5	4.4	0.7	19.3	35.7	0	11.4	13.0
Plateau	27.9	2.6	1.5	14.6	32.5	1.6	9.8	9.5
Total	19.9	3.0	1.1	18.1	42.6	0.8	5.8	8.8

Source: The Challenge Initiative Background Household Survey, 2019.

Although PPMVs serve as a major source of primary health care commodities and FP services, they cannot provide long-acting or permanent methods. The survey found that male condoms were the most common contraceptive method obtained from PPMVs across the states, followed by emergency contraception (Table 3). Across the 4 states, almost 86% of respondents aged 15–19 years and 82% of respondents aged 20–24 years visited a PPMV to obtain a male condom. Approximately 17% of respondents aged 15–19 years and 19% of those aged 20–24 years across the 4 states obtained emergency contraception from PPMVs. Plateau State recorded the highest percentage of male condoms obtained by youth from the PPMVs irrespective of age, followed by Edo and then Ogun states. Equal proportions of respondents aged 20–24 years in Niger, Ogun, and Plateau states obtained oral contraceptive pills from PPMVs. When considering other short-acting methods across age groups, approximately 4% of respondents aged 20–24 years, compared to 1.4% of respondents aged 15–19 years, obtained injectables from PPMVs. Disaggregating by state showed that only respondents from Niger, irrespective of their age group, obtained injectables from PPMVs. However, less than 1% of youth aged 20–24 years in Edo and no one aged 15–19 years from any of the other states obtained injectables.

**TABLE 3.** Contraceptive Methods Currently Used by Youth and Adolescents and Obtained From Proprietary Patent Medicine Vendors, by State

	**State**									
	**Edo** **(N=177)**		**Niger** **(N=88)**		**Ogun** **(N=94)**		**Plateau** **(N=95)**		**Total** **(N=454)**	
	**15–19 Years,** **(n=63)**	**20–24 Years** **(n=114)**	**15–19 Years (n=24)**	**20–24 Years** **(n=64)**	**15–19 Years (n=27)**	**20–24 Years** **(n=67)**	**15–19 Years (n=31)**	**20–24 Years** **(n=64)**	**15–19 Years** **(n=145)**	**20–24 Years** **(n=309)**
Implant	0.0	0.0	0.0	0.0	0.0	0.0	0.0	0.0	0.0	0.0
Intrauterine device	0.0	0.0	0.0	0.0	0.0	0.0	0.0	0.0	0.0	0.0
Injectables	0.0	0.9	8.3	12.5	0.0	1.5	0.0	3.1	1.4	3.9
Sayana Press	0.0	0.0	0.0	0.0	0.0	0.0	0.0	0.0	0.0	0.0
Oral contraceptive pills	1.6	0.0	0.0	4.7	3.7	4.5	3.2	4.7	2.1	2.9
Emergency contraception	22.2	17.5	20.8	37.5	7.4	11.9	9.7	9.4	16.6	18.8
Male condom	82.5	90.4	79.2	64.1	85.2	82.1	96.8	90.6	85.5	81.8
Female condom	1.6	0.0	0.0	1.6	3.7	0.0	0.0	0.0	1.4	0.3
Standard days method/cycle bead	0.0	2.6	0.0	0.0	0.0	0.0	0.0	3.1	0.0	1.6
Lactational amenorrhea method	0.0	0.0	0.0	0.0	3.7	0.0	0.0	0.0	0.7	0.0

Source: The Challenge Initiative Background Household Survey, 2019.

PPMVs serve as a major source of primary health care commodities and FP services, offering convenience and confidentiality to adolescents and youth seeking contraception and FP services.

Concerns have been raised about PPMVs’ quality of services and their need to better comply with government regulations.^17^ Although Nigeria has policies in place that are meant to ensure that the public health system maintains its stewardship and regulatory role for performance and standards,^18^^–^^21^ a systematic review^17^ noted that many PPMVs operate without a proper license, stock drugs that they are not allowed by law to sell, provide diagnostic and treatment services that are outside of their scope, and have low rates of referrals to PHCs. Two studies cited in the review found that PPMV staff had high levels of awareness of contraceptive methods, but no studies assessed their knowledge of the proper use or the benefits and risks of different contraceptive methods. The sheer overwhelming availability and accessibility of PPMVs and the observed utilization of their services by many Nigerians, especially adolescents and youth, made it timely and strategic for Nigeria to harness PPMVs’ role within the Health Sector Strategy toward achieving universal health coverage and the Sustainable Development Goals set forth by the United Nations.^22^^,^^23^

We describe how 4 state governments in Nigeria were supported to develop a model to strengthen public-private partnerships between PPMVs and PHCs to leverage PPMVs to provide adolescents and youth with high-quality contraceptive information, services, and referrals to PHCs.^24^ The model aimed to ensure PPMVs’ understanding of and compliance with the national guidelines for FP^4^^,^^25^^,^^26^ and youth-friendly services, improve the coordination process by regular state-government-led supervision and coaching of PPMVs to improve their record-keeping and referral systems, and, ultimately, contribute to increasing contraceptive access and uptake for adolescents and youth.

## STRENGTHENING PROPRIETARY PATENT MEDICINE VENDORS TO PROVIDE CONTRACEPTIVE SERVICES

The overwhelming practice of adolescents and youth accessing contraceptives from private sector sources, especially the PPMVs, underscored their significance as health care providers because almost half of the survey respondents reported using PPMVs.

**FIGURE 1:**
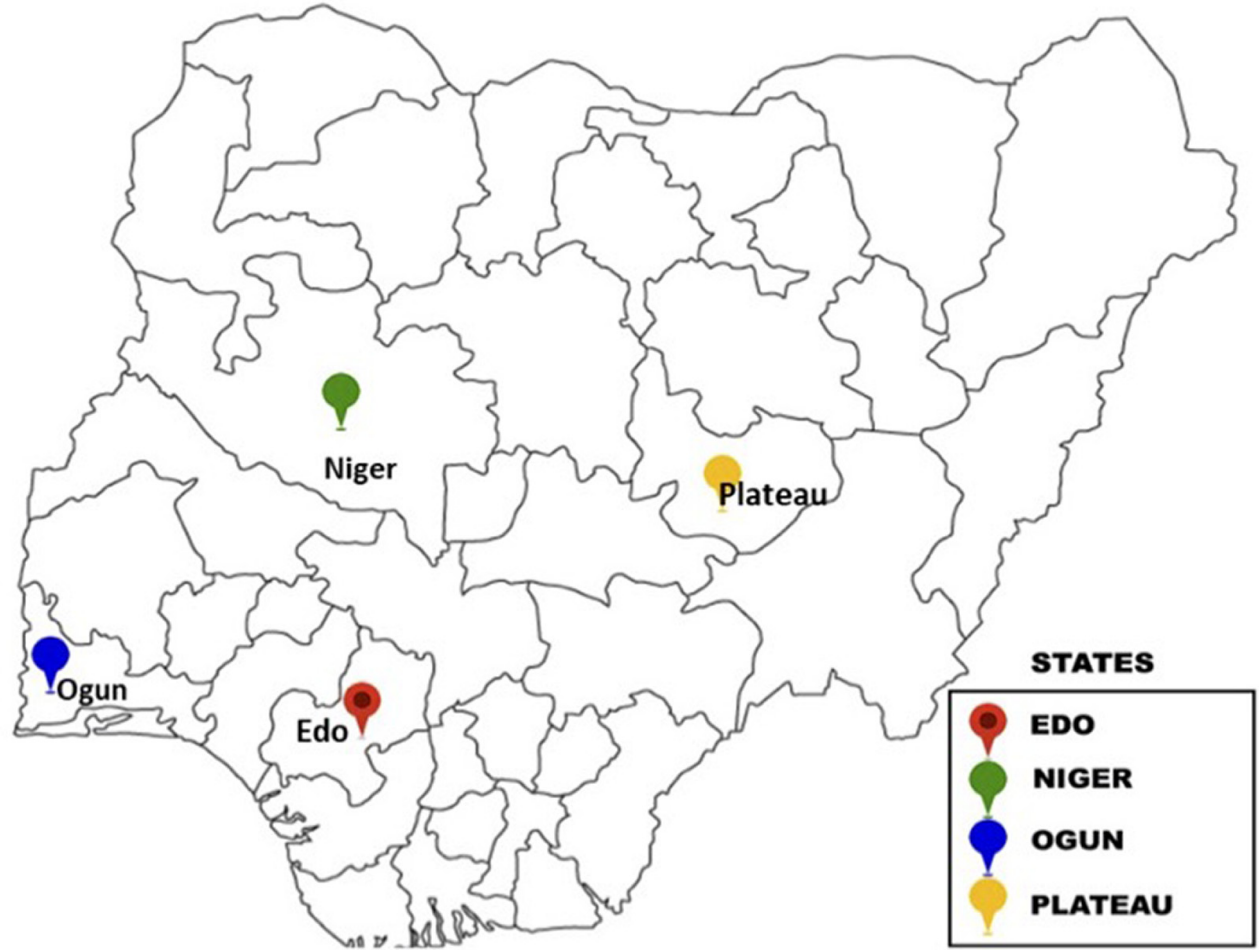
Map of Nigeria Showing Implementing States for the Adolescent and Youth Sexual and Reproductive Health Program

The Challenge Initiative (TCI) had already been working in Niger and Ogun states in Nigeria to improve contraceptive access women of reproductive age (WRA, aged 15–49 years), and in 2018, expanded its focus to married and unmarried adolescents and youth (aged 15–24 years).^24^ In 2019, TCI expanded into Edo and Plateau states (Figure 1). TCI’s overall goal was to increase modern contraceptive use among adolescents and youth aged 15–24 years by supporting increased intention to use modern contraception, delaying the age of first birth, and increasing healthy timing and spacing of pregnancy among adolescents and youth aged 15–24 years in urban poor cities.

As an important component of its AYSRH approaches, TCI Nigeria included a clear focus on building a collaborative partnership between PPMVs and PHCs. The process-related outcomes were to improve PPMVs’ compliance with national guidelines to ensure quality services and increase PPMV client referrals to PHCs for comprehensive sexual and reproductive health services. This would expand access to a full range of quality contraceptive services for adolescents and youth aged 15–24 years and increase contraceptive uptake through access to additional methods not dispensed by PPMVs.

### Hub-Spoke Model of Implementation

To strengthen the linkages between PPMVs and PHCs in delivering contraceptive services to adolescents and youth, TCI Nigeria supported the State Ministry of Health in establishing a hub-spoke model. This model focused on enhancing public-private collaboration, improving PPMVs’ adherence to the national guidelines of service quality, strengthening the referral system between PPMVs and PHCs, and maximizing the accessibility of adolescents and youth to all contraceptive methods. High-volume PHCs acted as hubs and linked to neighboring high-volume PPMVs as the spokes. The high-volume PHCs had high caseloads for antenatal care, labor and delivery, childhood routine immunizations, and FP services. The neighboring high-volume PPMVs had the largest numbers of clients and typically served as the initial point of contact for adolescents and youth. PPMVs were to provide convenient access to accurate information, counseling, and various contraceptive methods in line with national guidelines and facilitate client referrals to PHCs for specialized and other needed contraceptive options that were not provided by PPMVs. The PHC hub offered a broader range of reproductive health services and ensured quality of care.

To strengthen the linkages between PPMVs and PHCs in delivering contraceptive services to adolescents and youth, TCI Nigeria supported the State Department of Health in establishing a hub-spoke model of implementation.

The model was implemented between October 2019 and August 2021 through the following steps.

### Implementation Steps

#### Step 1: Introduction of Interventions to State Governments

In 2018, 7 Nigerian states expressed interest in participating in the TCI AYSRH program. However, only 4 states were selected based on specific criteria: political commitment, system readiness, resource contribution, potential impact size, and the involvement of members from the program design and implementation team. The partnership between TCI and these selected states was established within the State Ministry of Health and the Primary Health Care Development Agency. The coordination was carried out through the Adolescent Health and Development (AHD) unit. This partnership spanned between 2 to 3 years, depending on the starting date: September 2018 or November 2019. During this period, the AHD units of the local government areas (LGAs) in these 4 states collaborated with the PPMV associations to identify PPMVs for the intervention.

#### Step 2: Selection of Proprietary Patent Medicine Vendors

AHD unit officers of LGAs, with support from PPMV associations, selected PPMVs that had a high volume of clients, were registered with the government agency responsible for registration, and were within the catchment areas of high-volume PHC hubs. With the support of the PPMV professional associations, 193 PPMVs (spokes) were selected within the catchment communities of 130 high-volume PHCs (hubs) across 25 urban LGAs in Edo, Niger, Ogun, and Plateau states to take part in this PPMV–PHC partnership intervention. These PPMVs were linked to the PHCs to expand access to AYSRH services for adolescents and youth beyond what PPMVs could provide.

#### Step 3: Capacity-Building of Proprietary Patent Medicine Vendors

After selecting PPMVs, TCI Nigeria supported the State Ministry of Health and State Primary Health Care Development Agency through the AHD unit to build the capacity of PPMVs using whole-site orientations (WSOs), on-the-job training, and supportive supervision. These interventions equipped PPMV providers with an understanding and appropriate information on AYSRH and improved the quality of services provided. The AHD unit carried out the interventions in collaboration with the LGA reproductive health (RH) coordinators and executives of the PPMV associations at the local government level.

WSO is a cost-effective approach to orient all clinical and nonclinical staff on quality FP and AYRSH counseling, including informed choice, the full range of contraceptive options, and the unique sexual and reproductive health needs of adolescents and youth (Box 1, Table 4).^19^^–^^21^ The PPMV operators were also oriented on the national guidelines to improve their knowledge, attitudes, and skills on how to deliver quality adolescent- and youth-friendly contraceptive services and refer adolescent clients to PHCs to increase access to additional contraceptive methods not dispensed by PPMVs. These orientations served the critical function of introducing PPMV and PHC staff, opening interactions between them, and fostering a sense of partnership among these health care cadres, as well as allowing PPMVs to view LGA staff as integral members of the business and not as “watchdogs.”

**TABLE 4.** Adolescent and Youth Focused Whole-Site Orientations of PPMVs and PHCs in Four States in Nigeria

**State**	**Local Government Areas Covered**	**Facilities Covered**	**PHC Staff Trained**	**PPMV Operators Trained**	**Whole-Site Orientations Conducted**
Edo	6	30	30	52	34
Niger	6	28	56	44	28
Ogun	9	47	60	45	40
Plateau	5	25	52	52	92
Total	26	130	198	193	194

Abbreviations: PHC, primary health center; PPMV, proprietary patent medicine vendor.

BOX 1 Operationalization of Whole-Site Orientations

**Facilitators:**

- Clinical staff orientation was conducted by trainers/facilitators from the family/local obstetrician and gynecologist society who had updated knowledge, had good communication skills, and could provide voluntary services.
- Nonclinical staff (e.g., ab technicians, support staff, sweepers, guards, and pharmacists) orientation was conducted by local government officers at the local government area.

**Place:** Facility premises were used to avoid staff travel and curtail expenses.

**Duration:** Three-hour orientation for clinical staff, followed by 3 hours for nonclinical staff (preferably held on the same day).

**Coverage:** In 4 states, a total of 194 whole-site orientations were conducted, training 193 proprietary patent medicine vendor operators and 198 primary health center staff (Table 4).

On-the-job training and coaching were introduced where there were gaps to address these and improve capacities or skills. This hands-on coaching involved demonstrating how to dispense contraceptives and document them daily for their records.

#### Step 4: Strengthening of Referral Linkages

**FIGURE 2:**
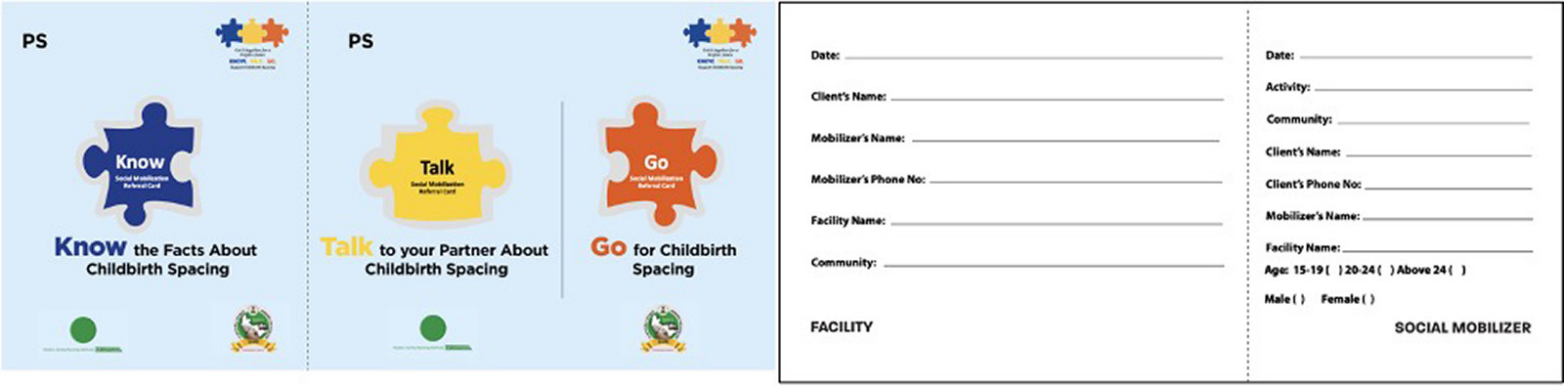
Front and Back of Go-Cards for Referral From Proprietary Patent Medicine Vendors to Primary Health Centers, Nigeria

The hub-spoke model aimed to strengthen the partnership between PPMVs and PHCs, leveraging PPMVs to provide high-quality contraceptive information, services, and referrals to PHCs for services beyond the scope of PPMVs through a systematic referral system by introducing referral cards known as “Go-Cards” at PPMVs (Figure 2). These cards were introduced to expand access to a range of services and methods for adolescents and youth. TCI coached staff of the State Ministry of Health and State Primary Health Care Development Agency (AHD and health educators) to introduce and orient PPMVs to use referral cards and document referrals through the LGA RH coordinators. Simple recording tools were introduced to ensure consistent and clear documentation of client information and medical history from PPMVs by the LGA RH coordinators. The process helped establish the importance of referring PPMV clients to PHCs for a full range of contraceptive services beyond what PPMVs could legally provide.

The completed referrals from the PPMVs were then counted at PHCs by the LGA supervisors. The Go-Card was introduced midway through the implementation process, but unfortunately, its purpose and documentation were recorded systematically in only Plateau state.

#### Step 5: Integration of Supportive Supervision and Coaching

Supportive supervision allows program managers to detect critical issues needing attention and to identify other capacity-building needs of service providers at the service points (clinical and nonclinical health facilities). In Nigeria, supportive supervision is integrated into quality improvement programs for public facilities in most states. Before this intervention, only government health facilities like PHCs received supportive supervision visits.^19^ TCI worked with partner states to include PPMVs systematically and intentionally in the supportive supervision visits (Box 2) to improve the quality of services at the PPMVs and strengthen provider skills.

BOX 2 Supportive Supervision for Proprietary Patent Medicine Vendors

The proprietary patent medicine vendor (PPMV) supervision team included 2–3 state officials with the following roles:

- Reproductive health coordinators observed compliance and quality of services
- Local government area monitoring and evaluation office documented and ensured data linkage to the primary health center hub
- Health educators followed up on Go-Card referrals

At each visit, the teams were responsible for the following:

- Interviewing the PPMV manager(s) and verifying service provision information in their records
- Recording observations on PPMV conditions (e.g., cleanliness, client seating available) on the PPMV facility observation checklist
- Verifying documentation of data on adolescent and youth client visits in the period under review by recording numbers from service registers/records, reviewing stock, and reconciling sales patterns with records.
- Noting the availability of information, education, and communications materials, including method leaflets, displayed to aid in information dissemination and facilitate uptake; visibility of condoms and emergency contraception pills; and availability of referral booklets (such as Go-Cards), as well as verifying documentation through review of records of activities.

PPMVs were systematically and intentionally included in supportive supervision visits to improve the quality of services at the PPMVs and strengthen provider skills.

**FIGURE 3:**
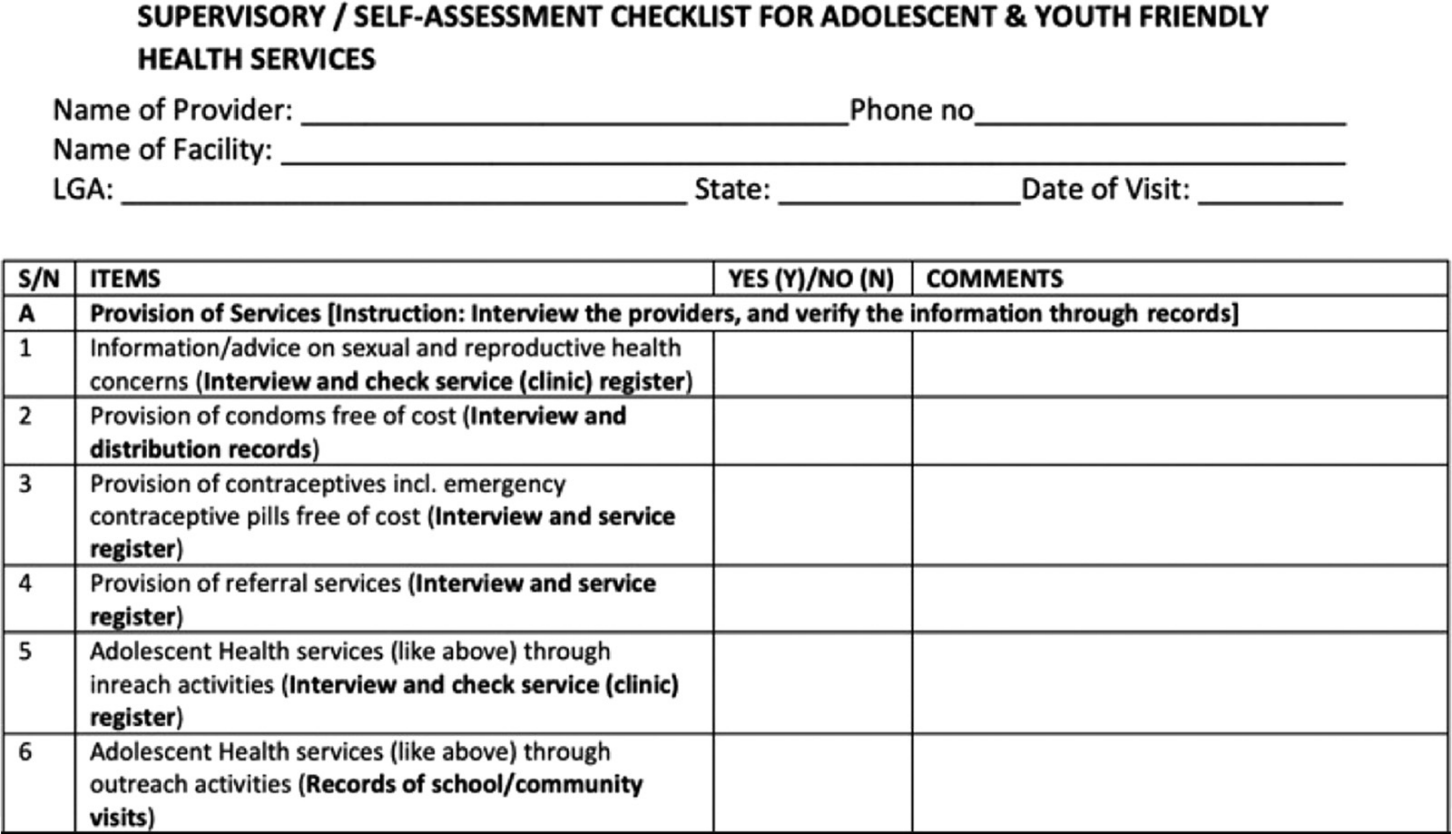
Compliance Checklist Used During Supervision Visits to Proprietary Patent Medicine Vendors, Nigeria

During their visits to PPMVs, the state supervision team used a compliance checklist (Figure 3) to ensure that PPMVs complied with national guidelines on the provision of quality FP services, documented services provided, and referred clients to PHCs for the services/methods that PPMVs were not allowed to provide. The supervision team visited each PPMV at least once per quarter for the period of their engagement. Each supervisory visit lasted for 3 hours on average.

The supervision team also provided PPMVs with ongoing coaching on FP/AYSRH counseling, client interaction, service provision, referrals, documentation, and tracking of age-disaggregated data. Coaching sessions were infused into the supervisor visits and organized follow-ups to the supervisory visits as needed. In each state, the coach collaborated with the state supervision team to identify gaps and areas where PPMVs needed assistance, as noted during supportive supervision. The coach documented the identified gap, action taken, and responsible person and used this information to plan the coaching session, decide with PPMVs whether it was to be virtual or in person, and agree upon a convenient coaching time.

These gaps were communicated to the relevant office/officers at the local government for follow-up. The state supervisory team members also functioned as coaches to make follow-up visits and provide technical assistance. The previous and latest supportive supervision tools and forms were compared to assess if the identified gaps had been addressed. Each PPMV received at least 3 rounds of supportive supervision within the intervention period, but the frequency of coaching depended on identified gaps and the extent of the problems identified. For example, to assist with documentation, TCI developed a simple, effective record-keeping system for PPMVs, which previously were not sufficiently tracking the services they provided or referrals made.

#### Step 6: Strengthening of Data Management

**FIGURE 4:**
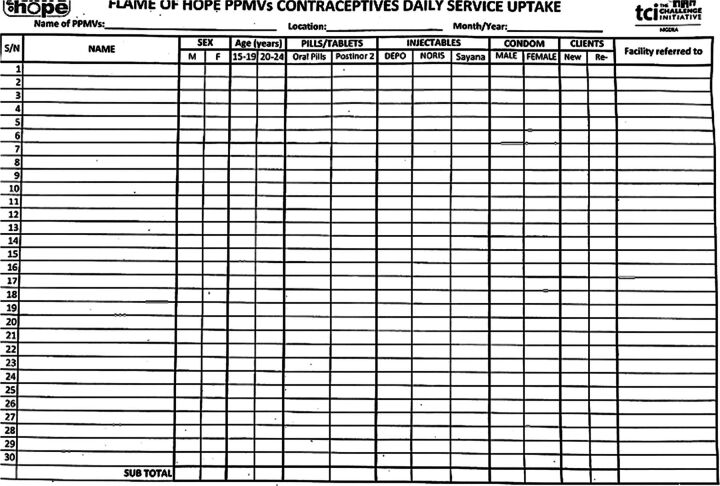
Form to Collect Monthly Service Provision Data From Proprietary Patent Medicine Vendors, Nigeria

After being trained on the newly designed government-TCI tool for age and method disaggregated client records (Figure 4), PPMVs began collecting data on service provision (e.g., age, pills, condoms, and emergency contraceptive pills dispensed and referrals made). The practice of collecting age-specific data was initiated with the support of the state government to PPMVs, which were not previously capturing such detailed client records.

Designated local government staff reviewed PPMV records before supervision visits and coaching were implemented and as the intervention was implemented. The data were shared with the LGA RH coordinator, who worked with service providers in the catchment PHC hub to input these data into the PHC registers because the PPMV data otherwise were not included in the HMIS. T-test analysis and distribution of statistics were done with the PPMV datasets.

Though PPMV staff were trained on referrals and consistently encouraged to refer clients to PHCs, the consistency of completing data recording on Go-Cards and tracking their deposits to PHCs was not enforced. However, during the last phase of the implementation, Plateau State strengthened the feedback system of documenting referrals from PPMVs to PHCs. As a result, referral data using Go-Cards were retrieved for Plateau State and showed that between July and September 2021, 797 clients (312 clients aged 15–19 years; 485 clients aged 20–24 years) were referred to PHCs for contraceptive uptake.

## LESSONS LEARNED

The PPMV-PHC partnership intervention is one of the approaches supported by TCI Nigeria’s AYSRH program, along with overall health systems strengthening, capacity-building, AYFHS facility-level quality improvements, and community-level social mobilization and demand generation like in-reaches and outreaches.^20^ These approaches are intended to ultimately result in increased access to and uptake of modern contraceptive methods among adolescents and youth aged 15–24 years in urban poor communities.

With proper orientation, a simple record-keeping system, and supportive supervision, PPMVs proved willing and able to document their age-disaggregated service provision, providing useful information that was added to the service registers at the PHCs. A total of 797 persons were referred through the PPMV-PHC referral in Plateau State within a period of 3 months.

### The Hup-Spoke Model Improves Access and Quality of Services

The establishment of a functional referral mechanism between PPMVs and PHCs allowed greater method choice for adolescents and youth, increased contraceptive uptake, more assurance that PPMVs were not offering contraceptive commodities and services they were not legally permitted to provide, and improved ability to monitor the referral linkages between PPMVs and PHCs.

However, we acknowledge that, of the 4 states in the intervention, only Plateau State was able to fully execute and document a referral system to PHCs. It is important to map PPMVs in relation to their proximity to PHCs offering adolescent- and youth-friendly health services for easy access, coaching support, and supply of commodities. For example, Edo State (which started the PPMV-PHC linkage later than the other states) had a wide catchment area between the spoke PPMVs and hub PHCs area, making it challenging to retrieve completed referrals from the PHCs due to transportation costs.

### Integrating The Private and Public Sectors Can Expand Access to Contraceptives

The presence of approximately 200,000 PPMVs in Nigeria in contrast to the 30,000 PHCs^3^^,^^4^ presented a tremendous opportunity to enhance the quality and availability of contraceptive services for individuals aged 15–24 years who seemed to prefer PPMVs for getting contraceptive methods. By strategically integrating PPMVs into the state FP/AYSRH program through targeted interventions, including capacity-building, supportive supervision, referral systems, and efficient data management, the government can significantly expand access to high-quality FP services. This intentional collaboration between PPMVs and PHCs, established at the grassroots level in local communities and LGAs, could create a powerful partnership. By leveraging this extensive network of PPMVs and linking them to high-volume PHCs in urban dense informal settlements, a diverse range of contraceptive methods tailored to the specific needs of adolescents and youth could be offered. Empowering this cohort with comprehensive choices and knowledge will not only improve their overall well-being but also promote the responsible use of contraceptives, ensuring a healthier future for the nation.

By strategically integrating PPMVs into the state FP/AYSRH program through targeted interventions, the government can significantly expand access to high-quality FP services.

### Improving the Quality of Services Can Contribute to Increased Contraceptive Uptake

Because the PPMV intervention was nested within the broader AYSRH approach of TCI Nigeria, we did not set out to assess the contribution of this intervention itself specifically on increased uptake of FP services and methods. However, the analysis of data records during early and intensified intervention periods for the PHC-PPMV hub-spoke partnership showed that participating PPMVs recorded a 54% increase in contraceptives dispensed to adolescents and youth aged 15–24 years, from 25,267 to 46,463. This suggested that service quality improvement steps undertaken through WSOs and supportive supervision visits may have contributed to the increased numbers of contraceptives provided at participating PPMVs. WSOs and supportive supervision together positively increased skill-building opportunities among PPMVs and may have contributed to the improved counseling of young people on contraceptives, leading to increased referrals to the PHCs. During the intensified period of the intervention, PPMVs complied consistently with age-disaggregated documentation of their contraceptive service provision, whereas before the intervention, their record-keeping was sporadic at best and did not disaggregate client data by age. Despite the absence of incentives for PPMVs, their motivation stemmed from the capacity-building opportunities provided.

### Consistent Data Management Remains a Challenge

During the state’s routine supervisory visits to participating PPMVs, records of the contraceptive methods provided to individuals aged 15–24 years were noted to be documented in specific registers, which supported accountability on compliance with regulations and the monitoring of the PPMVs’ contraceptive services. Across the 4 states, data from the 193 participating PPMV records were retrieved by the LGA RH coordinators and transferred to the 130 hub PHCs to be included in their facility registers. Unfortunately, this process was not consistently maintained and remains challenging. Data collection was hindered by PPMVs’ reluctance to maintain records, and their exclusion from the state’s HMIS made it challenging to represent the contributions of PPMVs on state DHIS2.

Nevertheless, it shows the potential of including contraceptive data from PPMVs within the state HMIS at the PHC facility level, which was attested to by the state AHD officers, LGA RH coordinators, and health promotion officers who led the entire PPMV-PHC approach. Although these state officers were responsible for the integrated supportive supervision in clinical settings, sustaining this in nonclinical settings at the state and LGA levels remains a huge challenge. However, this is a possible area of research in the near future. Despite these hurdles, the potential for an effective partnership between PPMVs and PHCs is evident, with the capacity to expand access to contraceptive methods through a hub-spoke approach.

## CHALLENGES AFFECTING IMPLEMENTATION AND POTENTIAL SOLUTIONS

The early success of the PPMV-PHC partnership was noted, and our results show that the scale-up of PPMV-PHC linkages is justified. However, the absence of comparison sites posed challenges in establishing a direct connection between the quality improvement interventions at the PPMV level and the observed increases in contraceptives dispensed. Without comparison sites, it becomes difficult to determine if the observed changes are solely attributed to the interventions.

We faced some challenges that affected the originally anticipated timeline, which took between 13 months in some states and 22 months in others, and challenges remain to wide-scale application. We note the following implementation challenges and limitations and potential means of mitigating these challenges.

The profit-focused nature of PPMVs required costly staggered coaching sessions to allow them to attend to clients as well as strengthen their capacity. Flexible coaching methods, such as digital platforms and mobile apps, can accommodate the profit-driven schedules of PPMVs, reducing costs and ensuring training fits into their busy routines.

High staff attrition disrupted training continuity, impacting program effectiveness. Recognizing and rewarding experienced staff creates a stable environment for continuous learning and skill retention, mitigating the disruptive impact of high staff attrition.

Although the use of Go-Cards as a tracking and monitoring tool for effective referrals and linkages between PPMVs and PHCs is innovative, complete referral data were only available in Plateau state and only in the last 3 months of the intervention period. The limited availability of referral data further affected the attribution of the effectiveness and impact of the interventions. Simplifying data management through user-friendly digital tools and incentivized training can encourage accurate record-keeping.

Because the HMIS does not take data from PPMVs, we had to figure out an approach to documenting age-disaggregated data from the participating PPMVs, using an improvised means of bookkeeping to document contraceptives dispersed by age and ensuring that these data records were compiled, collated, and delivered physically in the monthly PHC registers. Advocate the deliberate inclusion of the PPMVs into state HMIS and DHIS 2 systems.

Strategic solutions are essential to address these challenges and ensure program success, with monitoring and programmatic adjustments as needed. A robust system of engaging in meaningful dialogue with PPMVs, understanding their challenges, and incorporating their feedback into the FP/AYSRH program could ensure that they remain responsive and tailored to their evolving needs.

## CONCLUSION

The PPMV intervention demonstrates the promise and potential of effective collaboration between PPMVs and PHCs—through integrated WSOs, government-led supportive supervision visits, referral systems, and efficient data management. This collaboration, in combination with TCI’s wider AYSRH programming, resulted in 797 adolescents and youth accessing a full range of contraceptive choices when referred to neighboring PHCs. Additionally, the significant increases observed in the number of contraceptives dispensed to adolescents and youth at participating PPMVs may indicate that the quality improvement steps implemented for the contraceptive services offered by PPMVs had a positive effect on increased numbers of adolescents and youth using the PPMV contraceptive services. Future triangulation with data from supervisory visits to quantify quality improvement could help explore and substantiate such linkages.

We recommend that the government should continue to strengthen the working relationship between PPMVs and PHCs to provide quality contraceptive information and services to young people living in urban poor communities and incorporate PPMVs into the routine supportive supervision of the state health system to ensure compliance and enhance the quality of the contraceptive services provided by the large number of PPMVs. In addition, program implementers should acknowledge the importance of a referral mechanism between PPMVs and PHCs and incorporate a referral scheme into the design and implementation of PPMV programs.

## Notes

**Peer Reviewed**

**First published online:** March 21, 2024.

## References

[1] World Health Organization (WHO). *Task Sharing to Improve Access To Family Planning/Contraception: Summary Brief*. WHO; 2017. Accessed February 27, 2024. https://www.who.int/publications/i/item/WHO-RHR-17.20

[2] High-Impact Practices in Family Planning (HIP). *Drug Shops and Pharmacies: Sources for Family Planning Commodities and Information*. United States Agency for International Development; 2013. Accessed February 27, 2024. https://www.fphighimpactpractices.org/wp-content/uploads/2022/06/DrugShops_2013.pdf

[3] National Population Commission (NPC); ICF International. *Nigeria Demographic and Health Survey 2013*. NPC/ICF International; 2014. Accessed February 27, 2024. http://dhsprogram.com/pubs/pdf/FR293/FR293.pdf

[4] National Population Commission (NPC); ICF. *Nigeria Demographic and Health Survey 2018*. NPC/ICF; 2019. Accessed February 27, 2024. https://www.dhsprogram.com/pubs/pdf/FR359/FR359.pdf

[5] ACTWatch. *Nigeria Outlet Survey Findings*. Population Services International; 2013. Accessed February 27, 2024. https://marketbookshelf.com/wp-content/uploads/2017/05/Nigeria-2013-outlet-survey-results.pdf

[6] Oye-Adeniran BA, Adewole IF, Umoh AV, et al. Sources of contraceptive commodities for users in Nigeria. PLoS Med. 2005;2(11):e306. 10.1371/journal.pmed.0020306. 16218768 PMC1255759

[7] Tetui M, Ssekamatte T, Akilimali P, et al. Geospatial distribution of family planning services in Kira Municipality, Wakiso District, Uganda. Front Glob Womens Health. 2021;1:599774. 10.3389/fgwh.2020.599774. 34816171 PMC8593998

[8] Health Policy Research Group conference presentations: Health System Global Conference 2022. Health Policy Research Group. Accessed February 27, 2024. https://hprgunn.com/hprg-conference-presentations-hsg-2022

[9] Matthews Z, Channon A, Neal S, Osrin D, Madise N, Stones W. Examining the “urban advantage” in maternal health care in developing countries. PLoS Med. 2010;7(9):e1000327. 10.1371/journal.pmed.1000327. 20856899 PMC2939019

[10] Loewenson R, Masotya M. Responding to Inequalities in Health in Urban Areas: A Review and Annotated Bibliography. EQUINET Discussion Paper 106. Training and Resource Support Center/Regional Network for Equity in Health in East and Southern Africa; 2015. Accessed February 27, 2024. https://www.equinetafrica.org/sites/default/files/uploads/documents/Diss_106_Ann_Bib_Urban_health_in_ESA_Dec2015.pdf

[11] Mberu BU, Haregu TN, Kyobutungi C, Ezeh AC. Health and health-related indicators in slum, rural, and urban communities: a comparative analysis. Glob Health Action. 2016;9(1):33163. 10.3402/gha.v9.33163. 27924741 PMC5141369

[12] Barnes J, Chandani T, Feeley R. *Nigeria Private Sector Health Assessment*. Abt Associates, Inc., Private Sector Partnerships-One; 2008. Accessed February 27, 2024. https://pdf.usaid.gov/pdf_docs/pnadz381.pdf

[13] Aregbeshola BS, Khan SM. Primary health care in Nigeria: 24 years after Olikoye Ransome-Kuti’s leadership. Front Public Health. 2017;5:48. 10.3389/fpubh.2017.00048. 28349050 PMC5346888

[14] Okonkwo AD, Okonkwo UP. Patent medicine vendors, community pharmacists and STI management in Abuja, Nigeria. Afr Health Sci. 2010;10(3):253–265. 21327137 PMC3035963

[15] Brieger WR, Osamor PE, Salami KK, Oladepo O, Otusanya SA. Interactions between patent medicine vendors and customers in urban and rural Nigeria. Health Policy Plan. 2004;19(3):177–182. 10.1093/heapol/czh021. 15070866

[16] Corroon M, Kebede E, Spektor G, Speizer I. Key role of drug shops and pharmacies for family planning in urban Nigeria and Kenya. Glob Health Sci Pract. 2016;4(4):594–609. 10.9745/GHSP-D-16-00197. 28031299 PMC5199177

[17] Beyeler N, Liu J, Sieverding M. A systematic review of the role of proprietary and patent medicine vendors in healthcare provision in Nigeria. PLoS One. 2015;10(1):e0117165. 10.1371/journal.pone.0117165. 25629900 PMC4309565

[18] Asogwa MNO, Odoziobodo SI. Public private partnership in the provision of health services for the Millennium Development Goals: the imperative need for optimizing the public-private mix. Eur Sci J. 2016;12(29):175. 10.19044/esj.2016.v12n29p175

[19] Umar K, Okafor JI. Public private partnership in Nigeria, an overview. Int J Soc Sci Humanit Rev. 2015;5(2):66–71. Accessed February 27, 2024. https://www.ijsshr.com/journal/index.php/IJSSHR/article/view/116/101

[20] Powell-Jackson T, Macleod D, Benova L, Lynch C, Campbell OMR. The role of the private sector in the provision of antenatal care: a study of Demographic and Health Surveys from 46 low‐ and middle‐income countries. Trop Med Int Health. 2015;20(2):230–239. 10.1111/tmi.12414. 25358532

[21] Hallo De Wolf A, Toebes B. Assessing private sector involvement in health care and universal health coverage in light of the right to health. Health Hum Rights. 2016;18(2):79–92. 28559678 PMC5394993

[22] World Health Organization (WHO). *The World Health Report: Health Systems Financing: The Path to Universal Coverage*. WHO; 2010. Accessed February 27, 2024. https://apps.who.int/iris/handle/10665/44371 10.2471/BLT.10.078741 PMC2878164 20539847

[23] The 17 goals. United Nations. Accessed February 27, 2024. https://sdgs.un.org/goals

[24] Bose K, Martin K, Walsh K, et al. Scaling access to contraception for youth in urban slums: The Challenge Initiative’s systems-based multi-pronged strategy for youth-friendly cities. Front Glob Womens Health. 2021;2:673168. 10.3389/fgwh.2021.673168. 34816226 PMC8597915

[25] Federal Government of Nigeria. Federal Ministry of Health. (FMOH). *Nigeria Family Planning Blueprint 2020-2024*. FMOH; 2020. Accessed February 27, 2024. https://ngfrepository.org.ng:8443/jspui/handle/123456789/3303

[26] Federal Government of Nigeria. Federal Ministry of Health (FMOH). *Nigeria Family Planning Blueprint (Scale-Up Plan)*. FMOH; 2014. Accessed February 27, 2024. https://ngfrepository.org.ng:8443/jspui/handle/123456789/3165

